# Clinical characteristics, predisposing factors and outcomes of *Enterococcus faecium* and *Enterococcus faecalis* bloodstream infection in adults: a single-center retrospective study of 294 cases

**DOI:** 10.3389/fcimb.2026.1870585

**Published:** 2026-09-30

**Authors:** Hua-Zheng Guo, Zhen-Chuan Xing, Ting Ao, Ming Hu, Peng Zhen

**Affiliations:** 1 Department of Infectious Disease, Beijing Luhe Hospital, Capital Medical University, Beijing, China; 2 Department of Pulmonary and Critical Care Medicine, Beijing Luhe Hospital, Capital Medical University, Beijing, China

**Keywords:** bloodstream infection, *Enterococcus faecalis*, *Enterococcus faecium*, mortality, prognostic factors

## Abstract

**Background:**

Enterococci are critical pathogens of hospital-acquired bloodstream infection (BSI), with *E. faecalis* and *E. faecium* as the predominant species. Their incidence has been increasing. This study compared clinical characteristics, predisposing factors, and in-hospital mortality between the two species to inform clinical management.

**Methods:**

This single-center retrospective study enrolled adult patients with enterococcal BSI. Demographics, comorbidities, prior exposures, pre-existing infections, and SOFA scores were collected. Multivariable logistic regression identified factors associated with *E. faecium* BSI (variables with *p* < 0.10 in univariate analysis were entered). Risk factors for mortality were analyzed using multivariable logistic regression with BIC-based optimal subset selection in the overall cohort and each species subgroup. A clinically prespecified model was adopted for sensitivity analysis in the overall cohort.

**Results:**

Of 294 enrolled patients, 185 (62.9%) had *E. faecium* and 109 (37.1%) had *E. faecalis*. The overall in-hospital mortality was 49.7%. Age ≥ 65 years (aOR = 1.71, 95% CI: 1.04–2.83, *p* = 0.035) and ICU admission (aOR = 1.75, 95% CI: 1.01–3.04, *p* = 0.045) were independently associated with *E. faecium* BSI. Although mortality was higher in the *E. faecium* group (53.0% vs. 44.0%), the difference did not reach statistical significance (*p* = 0.139). Age ≥ 65 years, pneumonia, and SOFA score were independent risk factors for mortality in the overall cohort and *E. faecium* subgroup (all *p* < 0.05). Only SOFA score was significant in the *E. faecalis* subgroup. Enterococcal species did not show a significant association with mortality after multivariable adjustment (aOR = 1.49, 95% CI: 0.46–4.79, *p* = 0.501).

**Conclusions:**

In adult enterococcal BSI, hospital-acquired cases predominated. *E. faecium* was the dominant species, with higher ICU admission rates and a greater proportion of elderly patients than *E. faecalis*, although no statistically significant difference in mortality was observed. In the overall cohort and both subgroups, mortality was independently associated with disease severity, while no significant association was found for species.

## 1 Introduction

Enterococci are major pathogens of hospital-acquired bloodstream infection (BSI). *E. faecalis* and *E. faecium* are the two most common causative species in clinical practice (Al Bshabshe et al., 2024; Scharloo et al., 2024). The incidence of enterococcal BSI has been rising due to broad-spectrum antibiotic use, increased invasive procedures, and more immunocompromised patients (Sörstedt et al., 2024; Zerbato et al., 2024). In Europe and the United States, enterococci have become the second most common pathogens among Gram-positive bacterial BSI (Scharloo et al., 2024). *E. faecalis* and *E. faecium* differ in their virulence factor profiles. *E. faecalis* typically harbors a broader array of tissue-invasive virulence factors, such as gelatinase (*gelE*) and cytolysin (*cyl*), which facilitate tissue invasion, immune evasion, and biofilm formation (Ch'ng et al., 2019; Șchiopu et al., 2023). In contrast, *E. faecium* predominantly carries adhesion and colonization associated genes, including *esp*, *acm*, and pilus operons, which are closely linked to its ability to colonize medical devices and persist in the hospital environment (Sun et al., 2025). These genotypic differences may translate into distinct clinical manifestations and infection sources. In terms of antimicrobial resistance, both species are intrinsically resistant to cephalosporins and aminoglycosides (low-level) and exhibit considerable tolerance to cell wall-active agents (Kristich et al., 2014). Regarding acquired resistance, *E. faecium* is particularly notable, with ampicillin resistance rates exceeding 87% and vancomycin resistance (primarily mediated by *vanA* genotype) being almost exclusively confined to this species (Cattoir and Leclercq, 2013; Kristich et al., 2014; Huh et al., 2023; Al Bshabshe et al., 2024; Sun et al., 2025). *E. faecalis* typically remains susceptible to ampicillin. Consequently, an empirical ampicillin-based regimen that is effective against *E. faecalis* is often ineffective against *E. faecium*. This may delay the initiation of appropriate targeted therapy for *E. faecium* infections, which some studies have proposed as one of the reasons for the higher mortality associated with *E. faecium* BSI compared with *E. faecalis (*Lupia et al., 2022; Al Bshabshe et al., 2024). Regarding prognosis, some studies have reported that the 30-day mortality of enterococcal BSI ranges from 20% to 30%. However, the difference in mortality between *E. faecium* and *E. faecalis* remains inconclusive (Suppli et al., 2011; Lupia et al., 2022; Scharloo et al., 2024). Some studies have reported similar mortality rates between the two species (Sörstedt et al., 2024; Scharloo et al., 2024). In contrast, others have found that *E. faecium* BSI is associated with higher mortality, particularly in patients with concurrent immunosuppression or hematological malignancies (Zerbato et al., 2024). Furthermore, risk factors for mortality may differ between pathogen subgroups. Factors such as age, underlying disease burden, disease severity (SOFA score), source of infection, and receipt of appropriate antimicrobial therapy have all been reported as independent prognostic factors (Suppli et al., 2011; Lupia et al., 2022; Scharloo et al., 2024).

However, the aforementioned evidence is predominantly derived from Western cohorts. Comparative data on BSI caused by *E. faecalis* and *E. faecium* in Chinese populations remain scarce. Accordingly, we performed a single-center retrospective study to compare the clinical characteristics, risk factors, and in-hospital mortality associated with these two species in Chinese adults, aiming to provide local evidence for early risk stratification and optimization of empirical antibiotic therapy.

## 2 Methods

### 2.1 Study population and objectives

This study was a single-center retrospective study. The study screened patients with *E. faecium* or *E. faecalis* BSI who were admitted to Beijing Luhe Hospital, Capital Medical University between January 2019 and December 2024. Inclusion criteria: (i): BSI was diagnosed according to the CDC/NHSN surveillance definition of healthcare-associated infection (Horan et al., 2008); (ii) At least one set of blood cultures positive for *E. faecium* or *E. faecalis*; (iii) Clinical signs of systemic infection (fever ≥38.0 °C, chills, unexplained leukocytosis/leukopenia, or hypotension); (iv) Clinician-confirmed enterococcal BSI documented in medical records; (v) Age ≥ 18 years. Exclusion criteria: (i) Age < 18 years old. All cases were independently reviewed by two infectious disease specialists to confirm the diagnosis of true BSI. For patients who experienced multiple episodes of enterococcal BSI during the same hospitalization, only the first episode was included in the analysis. The objective of this study was to characterize and compare the clinical characteristics, predisposing factors, and in-hospital mortality between *E. faecium* and *E. faecalis* BSI in adults. The study was approved by the ethics committee of Beijing Luhe Hospital (2025-LHKY-017-01).

### 2.2 Clinical data

This study collected data on demographic characteristics, underlying comorbidities, clinical features, and clinical outcomes. All data collection was anchored to the time of enterococcal BSI onset. Onset was defined as the date on which the first set of blood cultures was collected and subsequently yielded a positive result for *E. faecium* or *E. faecalis*. The variables collected included: (1) age, sex, and prolonged bedridden status; (2) underlying comorbidities: diabetes mellitus, hypertension, ischemic heart disease, chronic pulmonary disease, cerebrovascular disease, heart failure, chronic liver disease, chronic kidney disease, hematologic disease, immune-related disorders, and active malignancy; (3) prior exposures and procedures: history of broad-spectrum antibiotic use within 90 days, hospitalization within 90 days, intensive care unit (ICU) admission, invasive ventilation, central venous catheterization (CVC), continuous renal replacement therapy (CRRT), and surgical procedure. ICU admission was defined as any stay in the ICU during the index hospitalization preceding BSI onset. Invasive mechanical ventilation, CVC, CRRT, and surgical procedures were defined as interventions received at any time during the same hospitalization prior to BSI onset. (4) pre-existing infections prior to BSI: urinary tract infection, intra-abdominal infection, hepatic infection, biliary infection, pneumonia, skin and soft tissue infection (SSTI), and intestinal infection; (5) clinical presentation: Sequential Organ Failure Assessment (SOFA) score (calculated based on the worst physiological and laboratory data within 24 hours after BSI onset), and presence of septic shock; (6) microbiological feature: vancomycin-resistant enterococcus (VRE) status; (7) outcomes: in-hospital mortality, and time to death (days, among non-survivors only); (8) type of acquisition: hospital-acquired versus community-acquired infection.

### 2.3 Microbiological methods

At least one set of blood cultures was collected from patients with suspected bacteremia per standard clinical practice. Blood samples were cultured using the Bact/ALERT 3D automated system (bioMérieux, France). Enterococcal BSI was defined by isolation of *Enterococcus* spp. from one or more blood culture sets. Species identification and antimicrobial susceptibility testing were performed using the Vitek-2 Compact system (bioMérieux, France) and the AST-GP67 card, respectively. Results were interpreted following CLSI guidelines.

### 2.4 Statistical analysis

Continuous variables were first tested for normality using the Shapiro-Wilk test. Continuous variables following a normal distribution were presented as mean ± standard deviation, and comparisons between groups were performed using the independent samples t-test. Non-normally distributed continuous variables were expressed as median with interquartile range (IQR) and compared using the Mann-Whitney U test. Categorical variables were summarized as counts (percentages) and compared using the chi-square test or Fisher’s exact test, as appropriate.

To identify factors independently associated with *E. faecium* BSI (relative to *E. faecalis* BSI), bivariate analyses were first performed. Variables with a *p*-value < 0.10 in these comparisons were selected as candidates for subsequent multivariable logistic regression. All candidate variables were then entered simultaneously using the forced entry method (Enter method) to calculate adjusted odds ratios (aORs) with 95% confidence intervals (CIs) and corresponding *p*-values.

To identify factors associated with in-hospital mortality, separate analyses were performed for the overall cohort, the *E. faecium* subgroup, and the *E. faecalis* subgroup. For each group, univariate logistic regression was first conducted, and variables with a *p*-value < 0.10 were selected as candidates for further analysis. Subsequently, best subset selection based on the Bayesian Information Criterion (BIC) was applied to the candidate variables for each group to identify the optimal model. Finally, multivariable logistic regression was fitted using the variables identified by the BIC procedure, and adjusted odds ratios (aORs) with 95% CIs and corresponding *p*-values were calculated. To assess the robustness of the BIC-based model selection, particularly given the limited sample size in the *E. faecalis* subgroup, we performed a sensitivity analysis using a clinically prespecified model in the overall cohort. Based on prior literature and clinical plausibility, the following covariates were forced into the multivariable logistic regression model: age ≥ 65 years, *Enterococcus* species, SOFA score, pneumonia, diabetes mellitus, broad-spectrum antibiotic use within 90 days, CVC and hospital-acquired infection. SOFA score was entered into the regression model as a continuous variable, with the odds ratio (OR) reflecting the effect per 1-point increase. To test the linearity assumption of this variable, restricted cubic splines with four knots were applied in the overall cohort. Model discrimination was assessed using the area under the receiver operating characteristic curve (AUC), and multicollinearity was evaluated using the variance inflation factor (VIF), with VIF < 2 considered to indicate no significant collinearity. Additionally, to evaluate model calibration and correct for potential overfitting, all models were internally validated using 500 bootstrap resampling iterations, with calculation of the calibration slope and Brier score.

All data included were derived from the hospital information system (HIS). During the data extraction process, the completeness of all variables was rigorously verified, and no missing values were identified for any of the enrolled patients. Statistical analyses were performed using Free Statistics analysis platform (Version 2.4, Beijing, China). Statistical significance was set at a two-sided *p* < 0.05 for final model coefficients and primary comparisons.

## 3 Results

### 3.1 Clinical characteristics

A total of 298 patients with enterococcal BSI were initially screened. After excluding 4 patients aged < 18 years, 294 patients were finally enrolled. Among them, 109 (37.1%) had *E. faecalis* BSI (61 in the survival group and 48 in the non-survival group), and 185 (62.9%) had *E. faecium* BSI (87 in the survival group and 98 in the non-survival group) (**Figure 1**). The majority (66.3%, n = 195) were male, with a median age of 69.0 (IQR, 59.0–78.8) years. Notably, 63.9% (n = 188) of patients were aged ≥ 65 years. Hospital-acquired infection accounted for 77.2% (n = 227) of cases, while community-acquired infection accounted for 22.8% (n = 67). The all-cause in-hospital mortality rate was 49.7% (146/294) (**Table 1**; **Figure 2A**).

**Figure 1:**
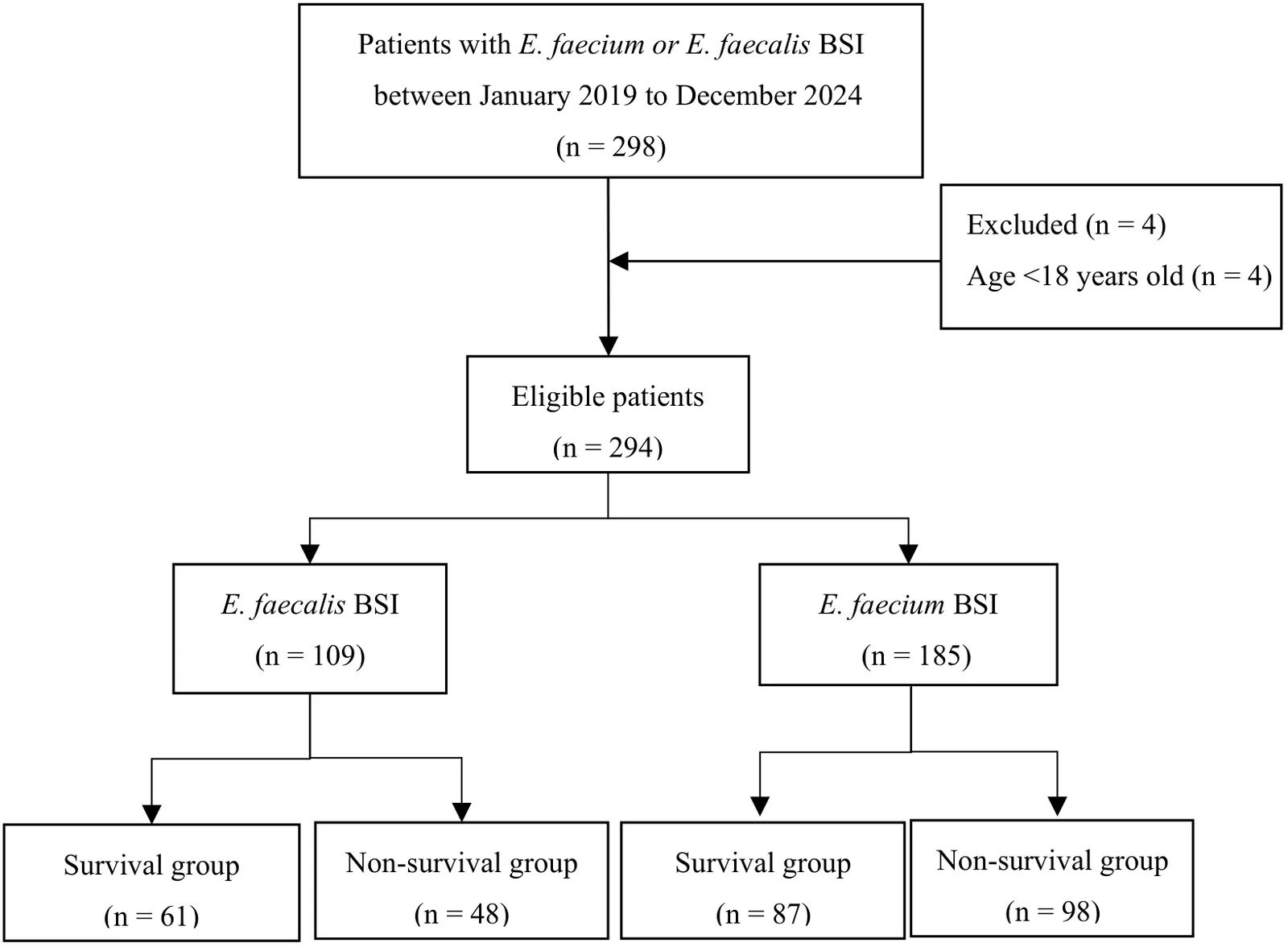
Patients flowchart.

**Table 1:** General characteristics and outcomes of all *E. faecium* and *E. faecalis* bloodstream infection.

Variable	All BSI (n = 294)	*E. faecium* BSI (n = 185)	*E. faecalis* BSI (n = 109)	*p*
Baseline characteristics				
Male, n (%)	195 (66.3)	120 (64.9)	75 (68.8)	0.490
Age, median (IQR)	69.0 (59.0, 78.8)	70.0 (60.0, 78.0)	69.0 (57.0, 79.0)	0.382
Age group, n (%)				0.053
< 65 years, n (%)	106 (36.1)	59 (31.9)	47 (43.1)	
≥ 65 years, n (%)	188 (63.9)	126 (68.1)	62 (56.9)	
Prolonged bedridden status, n (%)	29 (9.9)	18 (9.7)	11 (10.1)	0.920
Comorbidities, n (%)				
Diabetes mellitus	117 (39.8)	73 (39.5)	44 (40.4)	0.878
Hypertension	185 (62.9)	117 (63.2)	68 (62.4)	0.883
Ischemic heart disease	97 (33.0)	60 (32.4)	37 (33.9)	0.790
Chronic pulmonary disease	42 (14.3)	30 (16.2)	12 (11.0)	0.218
Cerebrovascular disease	107 (36.4)	68 (36.8)	39 (35.8)	0.866
Heart failure	71 (24.1)	39 (21.1)	32 (29.4)	0.109
Chronic liver disease	18 (6.1)	12 (6.5)	6 (5.5)	0.734
Chronic kidney disease	76 (25.9)	46 (24.9)	30 (27.5)	0.615
Hematologic disease	17 (5.8)	14 (7.6)	3 (2.8)	0.088
Immune-related disorders	16 (5.4)	9 (4.9)	7 (6.4)	0.570
Active malignancy	62 (21.1)	42 (22.7)	20 (18.3)	0.377
Prior exposures/procedures, n (%)				
Broad-spectrum antibiotics (≤ 90d)	228 (77.6)	149 (80.5)	79 (72.5)	0.109
Hospitalization (≤ 90d)	66 (22.4)	41 (22.2)	25 (22.9)	0.878
ICU admission	214 (72.8)	141 (76.2)	73 (67.0)	0.085
Invasive ventilation	191 (65.0)	126 (68.1)	65 (59.6)	0.141
CVC	210 (71.4)	131 (70.8)	79 (72.5)	0.760
CRRT	81 (27.6)	51 (27.6)	30 (27.5)	0.993
Surgical procedure	106 (36.1)	67 (36.2)	39 (35.8)	0.940
Pre-existing infections prior to BSI				
Urinary tract infection, n (%)	56 (19.0)	36 (19.5)	20 (18.3)	0.815
Intra-abdominal infection, n (%)	48 (16.3)	25 (13.5)	23 (21.1)	0.089
Hepatic infection, n (%)	6 (2.0)	6 (3.2)	0 (0.0)	0.088
Biliary infection, n (%)	33 (11.2)	24 (13.0)	9 (8.3)	0.216
Pneumonia, n (%)	147 (50.0)	96 (51.9)	51 (46.8)	0.398
SSTI, n (%)	30 (10.2)	17 (9.2)	13 (11.9)	0.454
Intestinal infection, n (%)	14 (4.8)	6 (3.2)	8 (7.3)	0.111
Clinical presentation				
SOFA score, median (IQR)	6.0 (3.0, 10.0)	7.0 (3.0, 10.0)	5.0 (3.0, 10.0)	0.154
Septic shock	142 (48.3)	88 (47.6)	54 (49.5)	0.744
Microbiology, n (%)				
VRE, n (%)	15 (5.1)	12 (6.5)	3 (2.8)	0.160
Outcomes				
In-hospital mortality, n (%)	146 (49.7)	98 (53.0)	48 (44.0)	0.139
Time to death (days) ^a^, median (IQR)	7.0 (3.0, 15.0)	7.0 (3.0, 15.0)	7.0 (3.0, 15.0)	0.867
Type of acquisition, n (%)				0.534
Hospital-acquired	227 (77.2)	145 (78.4)	82 (75.2)	
Community-acquired	67 (22.8)	40 (21.6)	27 (24.8)	

P-values compare E. faecium vs E. faecalis.

Continuous variables were compared using the independent samples t-test for normally distributed data and the Mann-Whitney U test for non-normally distributed data; categorical variables were compared using the chi-square or Fisher’s exact test.

a Among non-survivors only (n = 146).

ICU, intensive care unit; CVC, central venous catheterization; CRRT, continuous renal replacement therapy; SSTI, skin and soft tissue infection; SOFA, Sequential Organ Failure Assessment; VRE, vancomycin-resistant *enterococcus*.

**Figure 2:**
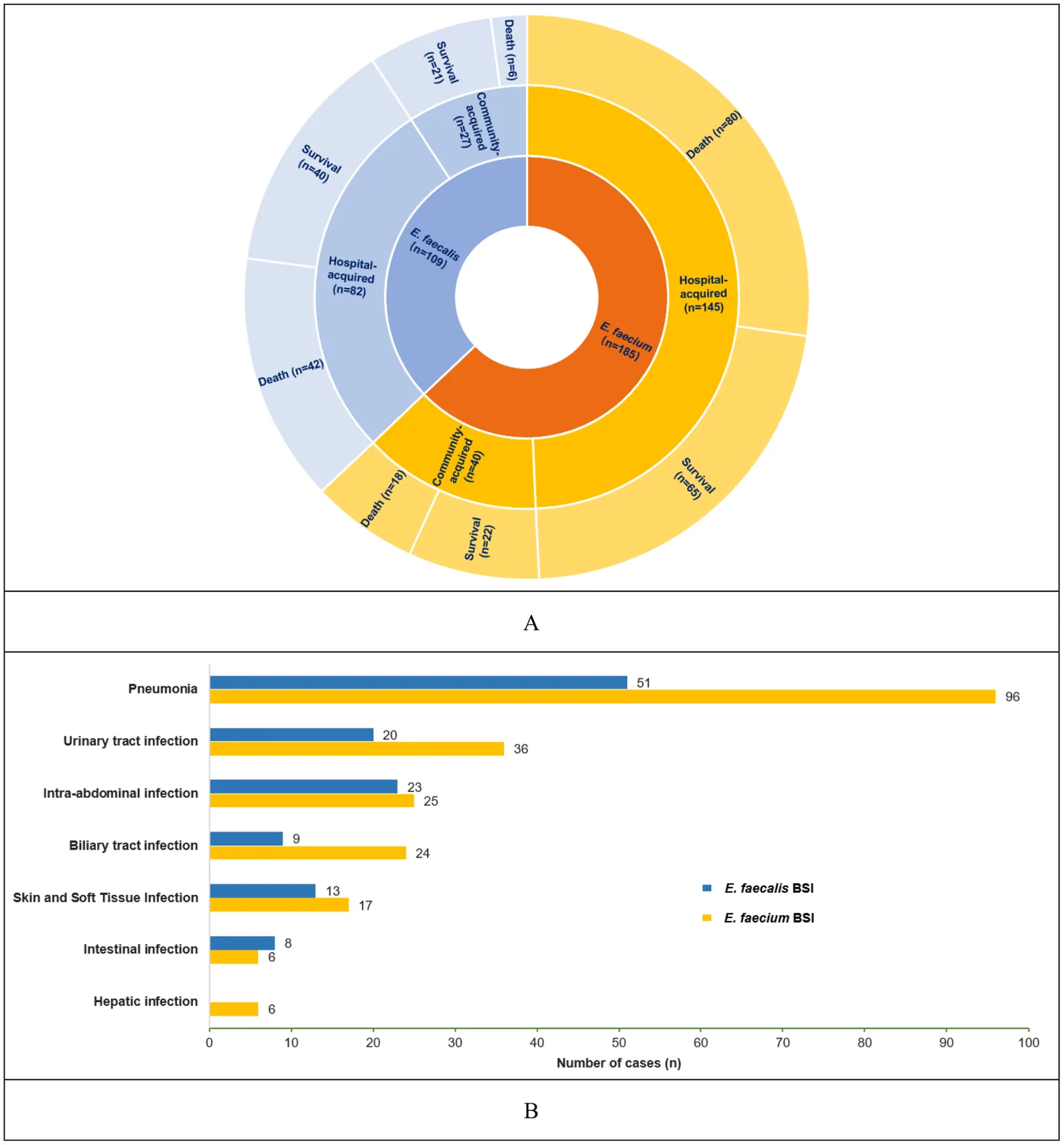
**(A)** Sunburst diagram of species distribution, type of acquisition, and clinical outcomes among 294 patients with *E. faecium* and *E. faecalis* BSI. **(B)** Bar chart of pre-existing infectious foci prior to BSI caused by *E. faecium* and *E. faecalis*.

### 3.2 Comparison between *E. faecium* and *E. faecalis* BSI groups

Bivariate comparisons between the two groups revealed no significant differences in sex, age (as a continuous variable), prolonged bedridden status, or most comorbidities (including diabetes mellitus, hypertension, ischemic heart disease, chronic pulmonary disease, cerebrovascular disease, heart failure, chronic liver disease, chronic kidney disease, immune-related disorders, and active malignancy) (all *p* > 0.05). The proportions of patients aged ≥ 65 years (68.1% vs. 56.9%, *p* = 0.053) and those with hematologic disease (7.6% vs. 2.8%, *p* = 0.088) were both higher in the *E. faecium* group than in the *E. faecalis* group, with borderline statistical significance. Regarding prior exposures, the ICU admission rate was higher in the *E. faecium* group than in the *E. faecalis* group, with borderline statistical significance (76.2% vs. 67.0%, *p* = 0.085). No significant differences were observed between the two groups in broad-spectrum antibiotic use within 90 days, hospitalization within 90 days, invasive ventilation, CVC, CRRT, or surgical procedures (all *p* > 0.05) (**Table 1**). With respect to pre-existing infections prior to BSI, intra-abdominal infection (21.1% vs. 13.5%, *p* = 0.089) and hepatic infection (3.2% vs. 0.0%, *p* = 0.088) showed borderline differences between the two groups, whereas no significant differences were observed in the distribution of other infection types (**Table 1**; **Figure 2B**). No significant differences were observed between the *E. faecium* and *E. faecalis* groups in SOFA score (7.0 vs. 5.0, *p* = 0.154) or the incidence of septic shock (47.6% vs. 49.5%, *p* = 0.744). Although the in-hospital mortality was numerically higher in the *E. faecium* group (53.0% vs. 44.0%), the difference did not reach statistical significance (*p* = 0.139). Vancomycin-resistant Enterococcus (VRE) accounted for 5.1% (15/294) of all isolates, with proportions of 6.5% (12/185) in the *E. faecium* group and 2.8% (3/109) in the *E. faecalis* group. Although the proportion of VRE was higher in the *E. faecium* group, the difference was not statistically significant (*p* = 0.160), possibly due to the small number of VRE cases (**Table 1**).

Variables with *p* < 0.10 in bivariate analysis (age ≥ 65 years, hematologic disease, ICU admission, intra-abdominal infection, and hepatic infection) were entered into multivariable logistic regression. Hepatic infection was excluded due to complete separation (no events in the *E. faecalis* group). The results showed that age ≥ 65 years (aOR = 1.71, 95% CI: 1.04–2.83, *p* = 0.035) and ICU admission (aOR = 1.75, 95% CI: 1.01–3.04, *p* = 0.045) were independently associated with *E. faecium* BSI. Hematologic disease demonstrated a borderline significant association (aOR = 3.49, 95% CI: 0.94–12.96, *p* = 0.061), whereas intra-abdominal infection did not (aOR = 0.66, 95% CI: 0.35–1.25, *p* = 0.204) (**Table 2**).

**Table 2:** Multivariable analysis of factors associated with *E. faecium* bloodstream infection (compared to *E. faecalis*).

Variable	aOR (95% CI)	*p*
Age ≥ 65 years	1.71 (1.04–2.83)	0.035
Hematologic disease	3.49 (0.94–12.96)	0.061
ICU admission	1.75 (1.01–3.04)	0.045
Intra-abdominal infection	0.66 (0.35–1.25)	0.204

aOR, adjusted odds ratio; CI, confidence interval; ICU, intensive care unit.

Variables with *p* < 0.10 in bivariate analysis were entered into multivariable logistic regression using the enter method. Hepatic infection was excluded due to complete separation (no events in the *E. faecalis* group).

### 3.3 Factors associated with all-cause in-hospital mortality

To evaluate disease severity across clinical outcome groups, SOFA scores were compared between survivors and non-survivors. In the overall cohort, the median SOFA score was 6.0 (IQR: 3.0–10.0), and was significantly higher among non-survivors than among survivors (10.0 [IQR: 8.0–12.0] vs. 3.0 [IQR: 1.0–4.0], *p* < 0.001). This disparity remained consistent across both enterococcal subgroups. In the *E. faecium* group, the median SOFA score was 7.0 (IQR: 3.0–10.0), and non-survivors exhibited significantly higher scores than survivors (10.0 [IQR: 8.0–12.0] vs. 3.0 [IQR: 2.0–4.0], *p* < 0.001). Similarly, in the *E. faecalis* group (median: 5.0 [IQR: 3.0–10.0]), non-survivors again had significantly higher scores than survivors (10.0 [IQR: 8.0–12.0] vs. 3.0 [IQR: 1.0–4.0], *p* < 0.001). Restricted cubic spline analysis was performed to evaluate the potential nonlinear relationship between SOFA score and clinical outcomes. The nonlinear component did not reach statistical significance (χ² = 4.80, df = 2, *p* = 0.091), supporting the inclusion of SOFA score as a continuous linear variable in subsequent regression models.

Bivariate analyses were first performed to identify factors associated with all-cause in-hospital mortality. In the overall cohort, factors significantly associated with increased in-hospital mortality included age ≥ 65 years, diabetes mellitus, ischemic heart disease, heart failure, broad-spectrum antibiotic use within 90 days, ICU admission, invasive ventilation, CVC, CRRT, pneumonia, SOFA score, septic shock, and hospital-acquired infection (all *p* < 0.05). In the *E. faecium* group, significant factors included age ≥ 65 years, ICU admission, invasive ventilation, CVC, CRRT, pneumonia, higher SOFA score, and septic shock (all *p* < 0.05). In the *E. faecalis* group, significant factors included age ≥ 65 years, ischemic heart disease, heart failure, chronic kidney disease, broad-spectrum antibiotic use within 90 days, ICU admission, invasive ventilation, CVC, CRRT, surgical procedure (protective factor), intra-abdominal infection (protective factor), pneumonia, higher SOFA score, septic shock, and hospital-acquired infection (all *p* < 0.05) (**Table 3**).

**Table 3:** Bivariate analysis of all-cause in-hospital mortality in *E. faecium* and *E. faecalis* bloodstream infection.

Variable	All BSI (n = 294)		*E. faecium* (n = 185)		*E. faecalis* (n = 109)	
	OR (95% CI)	*p*	OR (95% CI)	*p*	OR (95% CI)	*p*
Male, n (%)	1.01 (0.62–1.64)	0.968	0.86 (0.47–1.58)	0.629	1.41 (0.62–3.23)	0.412
Age ≥ 65 years, n(%)	2.13 (1.31–3.47)	0.002	1.87 (1.00–3.50)	0.049	2.43 (1.10–5.35)	0.028
Prolonged bedridden status, n (%)	1.28 (0.59–2.76)	0.532	1.12 (0.42–2.98)	0.817	1.60 (0.46–5.60)	0.462
Comorbidities, n (%)						
Diabetes mellitus	1.87 (1.16–3.00)	0.010	1.79 (0.98–3.26)	0.057	2.05 (0.94–4.46)	0.071
Hypertension	1.52 (0.94–2.45)	0.086	1.60 (0.88–2.92)	0.126	1.39 (0.63–3.05)	0.414
Ischemic heart disease	1.73 (1.06–2.83)	0.029	1.38 (0.74–2.57)	0.312	2.59 (1.15–5.85)	0.022
Chronic pulmonary disease	1.02 (0.53–1.95)	0.962	0.87 (0.40–1.90)	0.722	1.31 (0.39–4.35)	0.660
Cerebrovascular disease	1.50 (0.93–2.42)	0.097	1.20 (0.66–2.19)	0.546	2.19 (0.99–4.86)	0.054
Heart failure	1.93 (1.12–3.34)	0.018	1.36 (0.67–2.78)	0.399	3.54 (1.49–8.41)	0.004
Chronic liver disease	0.80 (0.31–2.09)	0.648	0.88 (0.27–2.84)	0.831	0.62 (0.11–3.53)	0.590
Chronic kidney disease	1.45 (0.86–2.46)	0.162	1.08 (0.55–2.10)	0.829	2.45 (1.04–5.79)	0.041
Hematologic disease	0.53 (0.19–1.48)	0.229	0.33 (0.10–1.09)	0.068	2.61 (0.23–29.66)	0.439
Immune-related disorders	0.78 (0.28–2.15)	0.628	1.12 (0.29–4.29)	0.874	0.49 (0.09–2.63)	0.403
Active malignancy	1.02 (0.58–1.78)	0.952	1.10 (0.55–2.19)	0.792	0.82 (0.30–2.19)	0.688
Prior exposures/procedures, n (%)						
Broad-spectrum antibiotics (≤ 90d)	2.84 (1.58–5.11)	< 0.001	1.76 (0.84–3.68)	0.133	5.97 (2.07–17.19)	0.001
Hospitalization (≤ 90d)	0.87 (0.50–1.51)	0.620	1.04 (0.52–2.08)	0.921	0.65 (0.26–1.63)	0.358
ICU admission	4.30 (2.42–7.63)	< 0.001	3.16 (1.54–6.47)	0.002	6.77 (2.51–18.26)	< 0.001
Invasive ventilation	3.76 (2.25–6.30)	< 0.001	3.17 (1.66–6.06)	< 0.001	4.79 (2.02–11.31)	< 0.001
CVC	3.21 (1.87–5.52)	< 0.001	3.14 (1.61–6.11)	0.001	3.55 (1.37–9.21)	0.009
CRRT	2.44 (1.43–4.16)	0.001	2.19 (1.12–4.29)	0.023	2.98 (1.24–7.12)	0.014
Surgical procedure	0.64 (0.39–1.03)	0.064	0.79 (0.43–1.44)	0.445	0.42 (0.18–0.96)	0.039
Pre-existing infections prior to BSI, n (%)						
UTI	0.59 (0.33–1.08)	0.086	0.65 (0.31–1.36)	0.255	0.48 (0.17–1.36)	0.167
Intra-abdominal infection	0.75 (0.40–1.40)	0.372	1.69 (0.71–4.05)	0.238	0.28 (0.09–0.82)	0.020
Hepatic infection	0.50 (0.09–2.77)	0.428	0.43 (0.08–2.42)	0.340	–	–
Biliary tract infection	1.25 (0.60–2.58)	0.552	0.87 (0.37–2.06)	0.755	2.76 (0.65–11.68)	0.167
Pneumonia	2.88 (1.79–4.62)	< 0.001	2.45 (1.36–4.43)	0.003	3.74 (1.68–8.30)	0.001
SSTI	0.65 (0.30–1.40)	0.267	0.77 (0.28–2.09)	0.609	0.53 (0.15–1.82)	0.310
Intestinal infection	1.37 (0.46–4.06)	0.567	4.62 (0.53–40.37)	0.166	0.75 (0.17–3.29)	0.700
Clinical presentation						
SOFA score	2.91 (2.25–3.75)	< 0.001	2.45 (1.90–3.16)	< 0.001	8.35 (2.37–29.49)	0.001
Septic shock	7.68 (4.58–12.9)	< 0.001	8.52 (4.36–16.62)	< 0.001	7.17 (3.05–16.84)	< 0.001
Microbiology, n (%)						
VRE	1.17 (0.41–3.31)	0.770	0.88 (0.27–2.84)	0.831	2.61 (0.23–29.66)	0.439
Type of acquisition, n (%)						
Hospital-acquired	2.08 (1.19–3.66)	0.011	1.50 (0.74–3.04)	0.255	3.67 (1.34–10.04)	0.011

Hepatic infection was excluded from the analysis due to complete separation (no events in the *E. faecalis* group). For variables where OR could not be estimated because of zero events in a specific subgroup, results are marked as “–”.

CRRT, continuous renal replacement therapy; CVC, central venous catheter; ICU, intensive care unit; SOFA, Sequential Organ Failure Assessment; SSTI, skin and soft tissue infection; UTI, urinary tract infection; VRE, vancomycin-resistant *Enterococcus*.

Variables with a *p*-value < 0.10 in the bivariate analysis were selected as candidates for multivariable logistic regression (the complete list of candidate variables is presented in **Table 3**). Best subset selection based on the Bayesian information criterion (BIC) was then applied to the candidate variables for each group to identify the optimal model, and the variables retained by the BIC procedure were subsequently entered into multivariable logistic regression models. Multivariable logistic regression showed that age ≥ 65 years, pneumonia, and higher SOFA score were independent predictors of in-hospital mortality in both the overall cohort (Model A) and the *E. faecium* subgroup (Model B) (all *p* < 0.05). In the *E. faecalis* subgroup (Model C), only higher SOFA score remained an independent predictor (aOR = 9.86 per 1-point increase, 95% CI: 2.14–45.41, *p* = 0.003), while no statistically significant difference was detected for diabetes mellitus (**Table 4**). Given the wide confidence intervals and the limited sample size of this subgroup, the finding should be interpreted with caution and considered exploratory. Model discrimination, calibration, and overfitting were evaluated using the AUC, calibration slope, and Brier score, with all metrics calculated from 500 bootstrap samples. Model A (overall cohort) achieved an AUC of 0.983 (95% CI: 0.970–0.995), with a calibration slope of 0.946 and a Brier score of 0.052. Model B (*E. faecium* subgroup) achieved an AUC of 0.970 (95% CI: 0.948–0.992), with a calibration slope of 0.933 and a Brier score of 0.073. Model C (*E. faecalis* subgroup) achieved an AUC of 0.997 (95% CI: 0.992–1.000), with a calibration slope of 0.968 and a Brier score of 0.027 (**Table 4**). In the overall cohort, a clinically prespecified multivariable logistic regression model (Model D) was constructed incorporating age, pneumonia, SOFA score, *Enterococcus* species, diabetes mellitus, CVC, hospital-acquired infection, and broad-spectrum antibiotic use within the preceding 90 days. After adjustment, age ≥ 65 years, pneumonia, and SOFA score remained independently associated with in-hospital mortality. In contrast, *E. faecium* (vs. *E. faecalis*), diabetes mellitus, CVC, hospital-acquired infection, and prior broad-spectrum antibiotic exposure did not show a significant association with mortality (all *p* > 0.05). The model demonstrated an AUC of 0.983 (95% CI: 0.970–0.997), with internal validation using 500 bootstrap resamples yielding a bias-corrected R² of 0.847, a calibration slope of 0.863, and a Brier score of 0.053 (**Table 4**). Across all models, all variance inflation factors for the included covariates were < 2.

**Table 4:** Multivariable logistic regression analysis of factors associated with in-hospital mortality.

Variable	aOR (95% CI)	*p*	AUC (95% CI)	Calibration slope	Brier score
Model A: All BSI (BIC-selected, n = 294)			0.983 (0.970–0.995)	0.946	0.052
Age ≥ 65 years	8.51 (2.41–30.04)	0.001			
Pneumonia	5.78 (1.89–17.72)	0.002			
SOFA score (per 1-point increase)	3.31 (2.41–4.54)	< 0.001			
Model B: *E. faecium* (BIC-selected, n = 185)			0.970 (0.948–0.992)	0.933	0.073
Age ≥ 65 years	5.17 (1.33–20.12)	0.018			
Pneumonia	4.11 (1.28–13.21)	0.018			
SOFA score (per 1-point increase)	2.66 (1.97–3.59)	< 0.001			
Model C: *E. faecalis* (BIC-selected, n = 109)			0.997 (0.992–1.000)	0.968	0.027
Diabetes mellitus	5.36 (0.29–99.78)	0.261			
SOFA score (per 1-point increase)	9.86 (2.14–45.41)	0.003			
Model D: All BSI (clinically prespecified, n = 294)			0.983 (0.970–0.997)	0.863	0.053
Age ≥ 65 years	9.11 (2.37–35.02)	0.001			
Pneumonia	6.02 (1.72–21.07)	0.005			
SOFA score (per 1-point increase)	3.37 (2.42–4.70)	< 0.001			
*E. faecium* (vs *E. faecalis*)	1.49 (0.46–4.79)	0.501			
Diabetes mellitus	1.87 (0.62–5.61)	0.264			
Central venous catheter	0.95 (0.18–5.08)	0.956			
Hospital-acquired infection	0.56 (0.10–3.27)	0.525			
Broad-spectrum antibiotics (≤ 90d)	3.48 (0.50–24.21)	0.208			

aOR, adjusted odds ratio; CI, confidence interval; SOFA, Sequential Organ Failure Assessment; AUC, area under the receiver operating characteristic curve; BSI, bloodstream infection; BIC, Bayesian Information Criterion.

SOFA score was entered as a continuous linear variable. Calibration slope and Brier score were calculated from 500 bootstrap resampling iterations; a calibration slope close to 1.0 indicates good calibration, and lower Brier scores indicate better predictive accuracy. All variance inflation factors for all variables in all models were < 2.

### 3.4 Vancomycin-resistant enterococci characteristics

VRE was detected in 15 of the 294 patients (5.1%), consisting of 12 *E. faecium* (6.5% of the *E. faecium* group) and 3 *E. faecalis* (2.8% of the *E. faecalis* group). All VRE cases were hospital-acquired (**Table 1**). The all-cause in-hospital mortality rate was 53.3% (8/15) in the VRE group, compared with 49.5% (138/279) in the vancomycin-susceptible group, though the difference did not reach statistical significance (*p* = 0.770). Given the small sample size, this result should be interpreted with caution. The antimicrobial susceptibility results for all VRE isolates are shown in **Figure 3**.

**Figure 3:**
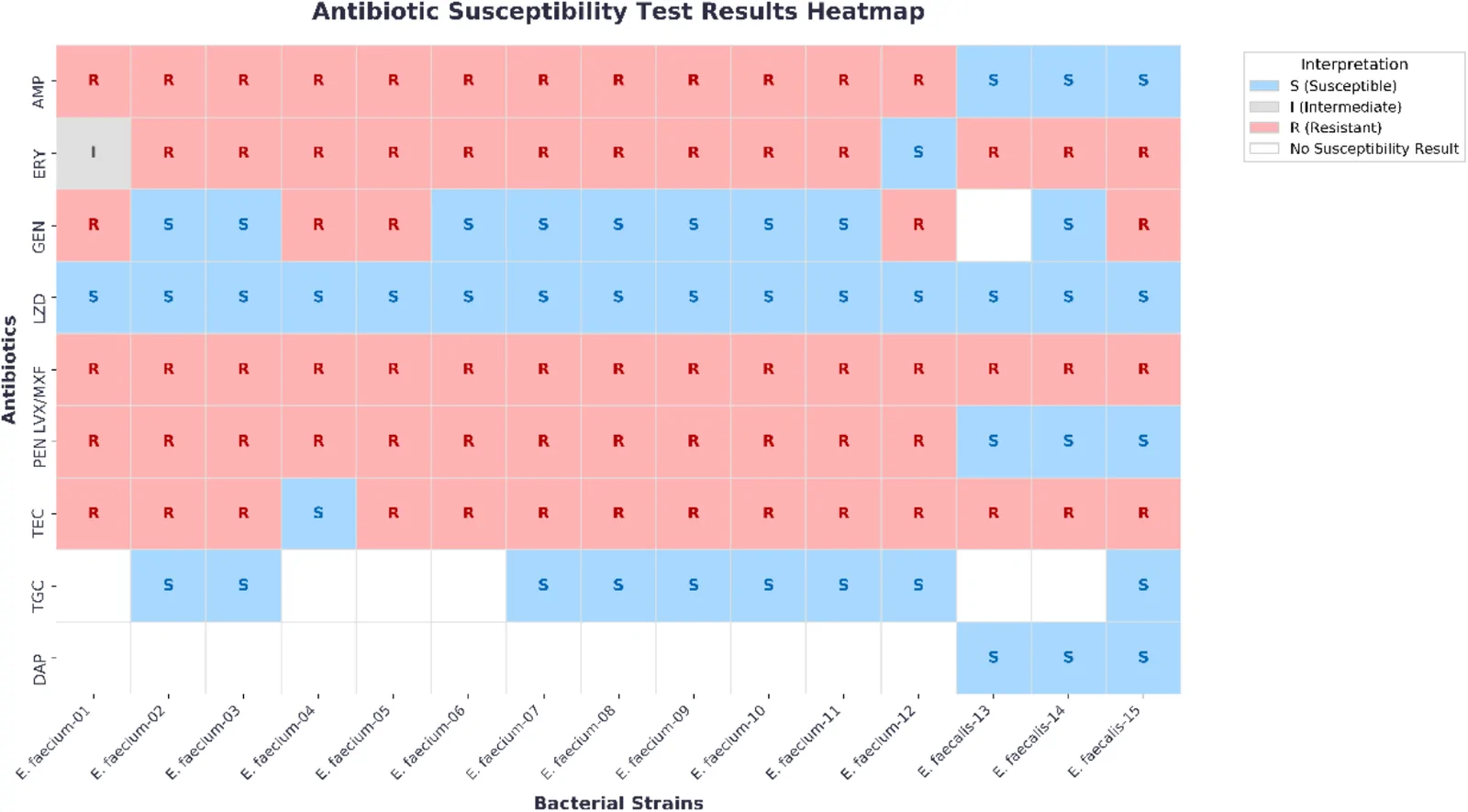
Antimicrobial susceptibility profiles of 15 VRE isolates. Color codes: blue = susceptible (S), gray = intermediate (I), red = resistant (R), white = no susceptibility result. AMP, Ampicillin; ERY, Erythromycin; GEN, High-concentration gentamicin; LZD, Linezolid; LVX/MXF, Levofloxacin/Moxifloxacin; PEN, Penicillin G, TEC, Teicoplanin; TGC, Tigecycline; DAP, Daptomycin.

## 4 Discussion

This study showed that adult enterococcal BSI were predominantly hospital-acquired (77.2%), with a predominance of elderly and male patients, and the majority had a history of healthcare exposures. *E. faecium* was the dominant species. Age ≥ 65 years (aOR = 1.71, 95% CI: 1.04–2.83, *p* = 0.035) and ICU admission (aOR = 1.75, 95% CI: 1.01–3.04, *p* = 0.045) were independently associated with *E. faecium* BSI. The overall all-cause in-hospital mortality rate was 49.7%. The in-hospital mortality rate was numerically higher in the *E. faecium* group than in the *E. faecalis* group (53.0% vs. 44.0%), although the difference did not reach statistical significance (*p* = 0.139). Of note, the significantly higher rates of ICU admission and proportion of elderly patients in the *E. faecium* group (all *p* < 0.05) further suggest that patients in this cohort had greater overall disease severity and poorer baseline status, indicating a trend toward worse prognosis. Multivariable analysis revealed that age ≥ 65 years, pneumonia, and SOFA score were independent risk factors for mortality in the overall population and *E. faecium* cohort (all *p* < 0.05), whereas in the *E. faecalis* cohort, only SOFA score was an independent risk factor. These findings may help guide risk stratification and antimicrobial stewardship.

In this study, *E. faecium* accounted for a higher proportion (62.9%), reflecting an important epidemiological shift in enterococcal infections. Traditionally, *E. faecalis* has been considered the predominant pathogen in enterococcal BSI. However, the proportion of *E. faecium* has been increasing globally over the past two decades (Hornuss et al., 2024; Scharloo et al., 2024). The German multicenter R-NET study reported that *E. faecium* accounted for 61% of Enterococcal BSI during 2016-2020 (Hornuss et al., 2024). Similarly, studies from the United Kingdom have shown that *E. faecium* has become the dominant species in enterococcal bacteremia (Horner et al., 2021; Hornuss et al., 2024). A Swedish study also demonstrated an increase in its proportion from 41% in 2015 to 51% in 2021 (Sörstedt et al., 2024). This shift may be associated with the enhanced acquired antimicrobial resistance of *E. faecium* and its advantage in transmission within hospital settings (Al Bshabshe et al., 2024; Scharloo et al., 2024). A 13-year retrospective study from China showed that *E. faecalis* remained the most common species (52.3%), although the proportion of *E. faecium* exhibited an increasing trend (Dai et al., 2022). Our study found that the proportion of *E. faecium* has markedly exceeded that of *E. faecalis*, which is consistent with recent findings from multiple countries.

The study observed no differences in comorbidities between *E. faecium* and *E. faecalis* BSI. Nevertheless, the prevalence of hematologic disease was higher in the *E. faecium* group, albeit without reaching statistical significance. A similar trend has been reported by Sörstedt et al. (2024). Further investigation with larger sample sizes is needed to validate the strength and clinical significance of this potential association. Patients exhibited a high burden of healthcare exposure, as previously reported. Both *E. faecalis* and *E. faecium* BSI occur mainly in critically ill patients with extensive medical interventions (Jin et al., 2025). Hospital-acquired accounted for 77%, suggesting that active prevention and control strategies targeting different exposure factors are needed. Regarding pre-existing infections, although enterococci are neither common pathogens nor colonizers of the respiratory tract, pneumonia prevalence was high in both groups. This probably reflects that our predominantly elderly and ICU-admitted population had poor baseline status and heavy healthcare exposure. Pneumonia may be a marker of these risk factors rather than a direct, species-specific source of enterococcal BSI. Urinary tract infection was the most common pre-existing infection in both groups (19.0%), suggesting it as a potential common source. *E. faecium* had higher proportions of hepatic and biliary infections, whereas *E. faecalis* had higher proportions of intra-abdominal and intestinal infections. Although not statistically significant, these differences may indicate a potential predisposition of the two species to colonize or invade distinct infection sites. Scharloo et al. reported that biliary and intra-abdominal sources were significantly associated with *E. faecium* BSI, while urinary tract sources were more common in *E. faecalis* BSI (Scharloo et al., 2024). Zheng et al. further confirmed that the two species possess distinct microbiological and clinical profiles, and should be considered separate clinical entities (Zheng et al., 2017). Both previous studies and the present study confirm species-specific differences in infection sources between *E. faecium* and *E. faecalis* BSI. Despite some inconsistency in specific findings, these differences underscore the need for further validation in larger cohort studies.

The in-hospital mortality rate in this cohort (49.7%) was substantially higher than the 20%–30% range previously reported in the literature (Suppli et al., 2011; Scharloo et al., 2024). This discrepancy may be attributable to multiple factors. First, patients in this cohort presented with high acuity of illness, as evidenced by an ICU admission rate of 72.8% prior to BSI onset, a shock rate of 48.3%, and a median SOFA score of 6.0, indicating a significant burden of organ dysfunction. Second, the proportion of elderly patients was high (62.9%), with a heavy comorbidity burden, among which diabetes mellitus (39.8%), ischemic heart disease (33.0%), and cerebrovascular disease (36.4%) were the most prevalent. Third, the proportion of hospital-acquired infections was extremely high (77.2%), and the majority of patients had extensive healthcare exposures, including antibiotic use within 90 days prior to infection (77.6%), central venous catheterization (71.4%), and surgical procedures (36.1%). Collectively, these factors explain why the mortality rate in our cohort exceeded that of previous reports, suggesting that our findings reflect the true disease burden in a critically ill tertiary care population. In subgroup comparisons, the crude mortality rate was higher in the *E. faecium* group than in the *E. faecalis* group, the difference did not reach statistical significance (53.0% vs. 44.0%, *p* = 0.139), which is consistent with the findings of the PROBAC study (Scharloo et al., 2024) and the meta-analysis by Eichel et al. (2023). Of note, patients in the *E. faecium* group had significantly higher rates of ICU admission (76.2% vs. 67.0%) and a greater proportion of elderly patients (≥ 65 years, 68.1% vs. 56.9%; all *p* < 0.05), suggesting that this subgroup had greater overall disease severity and poorer baseline health status. The lack of statistical significance in the between-group mortality difference may be partially constrained by insufficient statistical power, particularly given the limited sample size in the subgroups. Therefore, these results should be interpreted with caution and warrant further validation in larger prospective studies. Multivariable analysis identified age ≥ 65 years, pneumonia, and SOFA score as independent risk factors for mortality in the overall cohort and *E. faecium* subgroup, whereas only SOFA score in the *E. faecalis* subgroup. Jin et al. (2025) reported that a high Charlson Comorbidity Index (≥ 5), male, and delayed antimicrobial therapy were independent risk factors for mortality in ICU patients. Hemapanpairoa et al. (2021) confirmed that SOFA score was associated with 30-day mortality. Lupia et al. (2022) suggested that appropriate antimicrobial therapy alone does not improve outcomes in patients with multiple comorbidities, highlighting the need for comprehensive management strategies. Both previous studies and our results demonstrate that mortality risk is determined by underlying comorbidities, disease severity, and medical intervention intensity. The differences in mortality risk factors between the two species observed in our study may be due to the small sample size of the *E. faecalis* group (n = 109), resulting in insufficient statistical power. Alternatively, it may suggest that mortality risk in patients with *E. faecalis* BSI is primarily driven by the severity of acute illness rather than by age or metabolic comorbidities. Further validation in large-scale cohort studies is warranted.

In our study, the detection rate of VRE was 5.1%, with *E. faecium* accounting for 80%. A systematic review showed that VRE infection rates vary widely across different regions (1% - 55%), reaching 70% in vulnerable populations (Eichel et al., 2023). A relevant study from Croatia reported that the proportion of VRE increased from 12.7% in 2017 to 25.7% in 2021 (Todorić et al., 2023). These findings suggest that the epidemiological characteristics of VRE differ considerably across regions, populations. Multiple studies have indicated that VRE is an independent risk factor for mortality (Huh et al., 2023; Al Bshabshe et al., 2024; Zerbato et al., 2024). The lack of significance in our study may be attributable to the small sample size, and the results should be interpreted with caution. In addition, the VRE detection rate was numerically higher in the *E. faecium* subgroup than in the *E. faecalis* subgroup (6.5% vs. 2.8%), which is consistent with previous findings (Cattoir and Leclercq, 2013; Kristich et al., 2014; Huh et al., 2023; Sun et al., 2025) that *E. faecium* is more prone to acquired antimicrobial resistance. Although the difference between the two subgroups did not achieve statistical significance (*p* = 0.160), this result warrants cautious interpretation, particularly in light of the limited statistical power due to the small subgroup sample sizes.

This study has several limitations. First, it was a single-center, retrospective study, which may be subject to selection bias and information bias. The representativeness of a single-center sample for the epidemiological characteristics is limited, and the generalizability of the findings should be exercised with caution. Second, the overall sample size was relatively limited, particularly in the *E. faecalis* subgroup (n = 109, with 48 deaths). Due to the limited number of outcome events, the multivariable model in this subgroup yielded an excessively wide confidence interval for the SOFA score (aOR = 9.86, 95% CI: 2.14–45.41). This may be attributable to a quasi-separation phenomenon, reflecting a strong association between SOFA score and mortality. Consequently, the results of the subgroup analyses should be considered exploratory and interpreted with caution, and warrant further validation in larger cohort studies. Third, our study did not exclude cases of polymicrobial BSI, and the presence of co-pathogens may have influenced the clinical course and outcomes in a subset of patients. Although the exclusion of polymicrobial infections would be preferable for the precise evaluation of species‐specific effects of *Enterococcus*, in real‐world clinical practice, enterococcal BSI commonly occurs in critically ill patients presenting with multiple comorbidities or concomitant infectious foci. The primary exposure of interest in this study was the *Enterococcus* species. The current inclusion strategy may, in fact, mitigate artificial selection bias and thereby more faithfully reflect the real-world composition of the clinical population. Nevertheless, owing to the inherent constraints of the retrospective design, comprehensive data on polymicrobial infections were not available, precluding adjustment for polymicrobial infection as a covariate in the multivariable regression models. Future prospective studies incorporating stratified analyses according to monomicrobial versus polymicrobial infection status are warranted to more accurately delineate the species-specific effects of *Enterococcus*. Fourth, the use of broad-spectrum antibiotics accounted for 77.6%, representing a highly frequent exposure factor for enterococcal BSI. Owing to the limitations of the retrospective design, detailed information on the antibiotic exposure was unavailable. We were unable to analyze the association between exposure to specific antibiotic and the occurrence of enterococcal BSI or VRE. Fifth, this study presented the resistance profiles of 15 VRE isolates (5.1%). However, owing to the inherent limitations of the retrospective design and the unavailability of historical susceptibility records, we were unable to systematically provide comprehensive antimicrobial susceptibility data for VSE isolates. Future prospective studies with standardized susceptibility testing protocols are warranted to more comprehensively elucidate the association between species‐specific resistance and clinical outcomes. Sixth, we lacked detailed data on antimicrobial selection and treatment after the diagnosis of BSI. This may have affected the accuracy of our mortality risk assessment. Future multicenter, prospective, large-scale studies are warranted.

In conclusion, this study showed that adult enterococcal BSI were predominantly hospital-acquired (77.2%), predominantly affected elderly patients, and were associated with a substantial disease burden, with an overall in-hospital mortality rate as high as 49.7%. *E. faecium* was the dominant species, and the rates of ICU admission and proportion of elderly patients were significantly higher in the *E. faecium* group than in the *E. faecalis* group (all *p* < 0.05). However, no statistically significant difference in mortality was observed between the two subgroups (53.0% vs. 44.0%, *p* = 0.139), which may be attributable to the limited sample size and insufficient statistical power, and thus should be interpreted with caution. After adjusting for confounding factors, SOFA score remained significantly associated with mortality risk in the overall cohort (aOR = 3.31, 95% CI: 2.41–4.54, *p* < 0.001), whereas *Enterococcus* species (aOR = 1.49, 95% CI: 0.46–4.79, *p* = 0.501) was not significantly associated with in-hospital mortality. VRE accounted for 5.1% of isolates and were all hospital-acquired. No significant association was detected between VRE status and in-hospital mortality in this study. Given the limitations of the single-center retrospective design and the small subgroup sample sizes, the above findings warrant further validation in larger prospective studies.

## Data Availability

The original contributions presented in the study are included in the article/supplementary material. Further inquiries can be directed to the corresponding author.

## References

[B1] Al Bshabshe A. Algarni A. Shabi Y. Alwahhabi A. Asiri M. Alasmari A. . (2024). Characterization and antimicrobial susceptibility patterns of *Enterococcus* species isolated from nosocomial infections in a Saudi tertiary care hospital over a ten-year period (2012-2021). Diagnostics (Basel Switzerland) 14 (11), 1190. doi: 10.3390/diagnostics14111190 38893716 PMC11171566

[B2] Cattoir V. Leclercq R. (2013). Twenty-five years of shared life with vancomycin-resistant enterococci: is it time to divorce? J. Antimicrob. Chemother. 68, 731–742. doi: 10.1093/jac/dks469 23208830

[B3] Ch'ng J.-H. Chong K. K. L. Lam L. N. Wong J. J. Kline K. A. (2019). Biofilm-associated infection by enterococci. Nat. Rev. Microbiol. 17, 82–94. doi: 10.1038/s41579-018-0107-z 30337708 PMC13729018

[B4] Dai Z. Chen L. Y. Cai M. J. Yao Y. H. Zhu J. H. Fang L. L. . (2022). Clinical characteristics and microbiology of nosocomial enterococcal bloodstream infections in a tertiary-level hospital: a retrospective study, 2007-2019. J. Hosp. Infection 122, 203–210. doi: 10.1016/j.jhin.2022.01.011 35085678

[B5] Eichel V. M. Last K. Brühwasser C. von Baum H. Dettenkofer M. Götting T. . (2023). Epidemiology and outcomes of vancomycin-resistant *enterococcus* infections: a systematic review and meta-analysis. J. Hosp. Infection 141, 119–128. doi: 10.1016/j.jhin.2023.09.008 37734679

[B6] Hemapanpairoa J. Changpradub D. Thunyaharn S. Santimaleeworagun W. (2021). Does vancomycin resistance increase mortality? Clinical outcomes and predictive factors for mortality in patients with *Enterococcus faecium* infections. Antibiotics (Basel Switzerland) 10 (2), 105. doi: 10.3390/antibiotics10020105 33499102 PMC7911214

[B7] Horan T. C. Andrus M. Dudeck M. A. (2008). CDC/NHSN surveillance definition of health care-associated infection and criteria for specific types of infections in the acute care setting. Am. J. Infect. Control 36, 309–332. doi: 10.1016/j.ajic.2008.03.002 18538699

[B8] Horner C. Mushtaq S. Allen M. Hope R. Gerver S. Longshaw C. . (2021). Replacement of *Enterococcus faecalis* by *Enterococcus faecium* as the predominant enterococcus in UK bacteraemias. JAC-Antimicrob. Resist. 3, dlab185. doi: 10.1093/jacamr/dlab185 34909690 PMC8664539

[B9] Hornuss D. Göpel S. Walker S. V. Tobys D. Häcker G. Seifert H. . (2024). Epidemiological trends and susceptibility patterns of bloodstream infections caused by *Enterococcus* spp. in six German university hospitals: a prospectively evaluated multicentre cohort study from 2016 to 2020 of the R-Net study group. Infection 52, 1995–2004. doi: 10.1007/s15010-024-02249-2 38684586 PMC11499396

[B10] Huh K. Chung D. R. Ha Y. E. Ko J.-H. Huh H. J. Lee N. Y. . (2023). Impact of vancomycin resistance in *Enterococcus faecium* bloodstream infection on mortality: a retrospective analysis of nationwide surveillance data. Int. J. Infect. Dis. IJID Off. Publ. Int. Soc. For. Infect. Dis. 134, 8–14. doi: 10.1016/j.ijid.2023.04.411 37172775

[B11] Jin Y. Yi Z. Hu Y. Huang M. (2025). Epidemiology, clinical characteristics, and outcome of enterococcal bloodstream infections in critically ill patients in China: a single-center retrospective 10-year analysis. Microbiol. Spectr. 13, e0106725. doi: 10.1128/spectrum.01067-25 40788039 PMC12403588

[B12] Kristich C. J. Rice L. B. Arias C. A. (2014). “ Enterococcal infection—treatment and antibiotic resistance,” in Enterococci: From Commensals to Leading Causes of Drug Resistant Infection. Eds. Gilmore M. S. Clewell D. B. Ike Y. Shankar N. ( Massachusetts Eye and Ear Infirmary, Boston). Available online at: https://www.ncbi.nlm.nih.gov/books/NBK190420/ (Accessed September 26, 2026).

[B13] Lupia T. Roberto G. Scaglione L. Shbaklo N. De Benedetto I. Scabini S. . (2022). Clinical and microbiological characteristics of bloodstream infections caused by *Enterococcus* spp. within internal medicine wards: a two-year single-centre experience. Internal Emergency Med. 17, 1129–1137. doi: 10.1007/s11739-022-02926-w 35092582 PMC8799962

[B14] Scharloo F. Cogliati Dezza F. López-Hernández I. Martínez Pérez-Crespo P. M. Goikoetxea Aguirre A. J. Pérez-Rodríguez M. T. . (2024). Clinical characteristics, predisposing factors and outcomes for *Enterococcus faecalis* versus *Enterococcus faecium* bloodstream infections: a prospective multicentre cohort study. Eur. J. Clin. Microbiol. Infect. Dis. Off. Publ. Eur. Soc. Clin. Microbiol. 43, 2011–2022. doi: 10.1007/s10096-024-04917-5 39112668 PMC11405461

[B15] Șchiopu P. Toc D. A. Colosi I. A. Costache C. Ruospo G. Berar G. . (2023). An overview of the factors involved in biofilm production by the *Enterococcus* genus. Int. J. Mol. Sci. 24 (14), 11577. doi: 10.3390/ijms241411577 37511337 PMC10380289

[B16] Sörstedt E. Ahlbeck G. Snygg-Martin U. (2024). Trends in *Enterococcus faecium* bacteremia: exploring risk factors with emphasis on prior antibiotic exposure. Microorganisms 12 (10), 1932. doi: 10.3390/microorganisms12101932 39458242 PMC11509189

[B17] Sun H. Fang Y. Hu S. Lyu D. Dong X. Wang J. (2025). Molecular epidemiology, antimicrobial resistance, and virulence determinants of *Enterococcus faecium* bloodstream isolates: a 7-year study in Shandong, China. BMC Microbiol. 25, 765. doi: 10.1186/s12866-025-04510-6 41257595 PMC12628980

[B18] Suppli M. Aabenhus R. Harboe Z. B. Andersen L. P. Tvede M. Jensen J.-U. S. (2011). Mortality in enterococcal bloodstream infections increases with inappropriate antimicrobial therapy. Clin. Microbiol. Infection Off. Publ. Eur. Soc. Clin. Microbiol. Infect. Dis. 17, 1078–1083. doi: 10.1111/j.1469-0691.2010.03394.x 20946408

[B19] Todorić Z. Majdandžić I. Keretić Kregar T. Herljević Z. Ćorić M. Lešin J. . (2023). Increasing trend in enterococcal bacteraemia and vancomycin resistance in a tertiary care hospital in Croatia, 2017-2021. Infect. Dis. (London England) 55, 9–16. doi: 10.1080/23744235.2022.2131901 36240424

[B20] Zerbato V. Pol R. Sanson G. Suru D. A. Pin E. Tabolli V. . (2024). Risk factors for 30-day mortality in nosocomial enterococcal bloodstream infections. Antibiotics (Basel Switzerland) 13 (7), 601. doi: 10.3390/antibiotics13070601 39061283 PMC11273391

[B21] Zheng J.-X. Li H. Pu Z.-Y. Wang H.-Y. Deng X.-B. Liu X.-J. . (2017). Bloodstream infections caused by *Enterococcus* spp: a 10-year retrospective analysis at a tertiary hospital in China. J. Huazhong Univ. Sci. Technology. Med. Sci. = Hua Zhong Ke Ji Da Xue Xue Bao. Yi Xue Ying Wen Ban = Huazhong Keji Daxue Xuebao. Yixue Yingdewen Ban 37, 257–263. doi: 10.1007/s11596-017-1725-9 28397040

